# Metagenome-assembled genomes of two salt-tolerant methylotrophs enriched from a sulfur-rich Zodletone spring in Oklahoma, USA

**DOI:** 10.1128/mra.00461-25

**Published:** 2025-06-18

**Authors:** Imam Alam, Fares Najar, Babu Fathepure

**Affiliations:** 1 Department of Microbiology and Molecular Genetics, Oklahoma State University 7618, Stillwater, Oklahoma, USA; 2 High Performance Computing Center, Oklahoma State University 7618, Stillwater, Oklahoma, USA; Montana State University, Bozeman, Montana, USA

**Keywords:** metagenome-assembled genomes, methanotrophs, enrichment, saline environment

## Abstract

We obtained metagenome-assembled genomes (MAGs) of two salt-tolerant methylotrophic bacteria, *Methylohalobius* sp. strain ZOD2 and *Methyloligella* sp. strain ZOD6, from an enrichment culture derived from sediment collected at a sulfur-rich spring in Oklahoma, USA. These MAGs offer insights into the methane oxidation capabilities of these bacteria under high-salinity conditions.

## ANNOUNCEMENT

Methane mitigation remains a critical challenge, particularly from produced water storage tanks (PWST) and abandoned oil and gas (AOG) wells, where saline conditions hinder microbial methane oxidation (1). Despite the significant amount of methane being produced in PWSTs and AOGs, not much is known about the identity of methanotrophic microbial communities under saline conditions. To understand salt-tolerant methanotrophs, aerobic enrichment was initiated in 160 mL serum bottles containing 45 mL of mineral salts medium amended with 2.5 M NaCl (2) and inoculated with 5 g of sediment from the Zodletone spring (35°00′08.8″N 98°41′17.4″W). Bottles were closed with rubber septa and aluminum crimps and injected with 1% (vol/vol) methane in the headspace and incubated at 30°C. Oxidation of methane was monitored using a gas chromatograph (2). A stable enrichment was obtained after repeatedly feeding the culture with methane and periodically transferring 50% of the culture to fresh medium for over 3 months.

**Fig 1:**
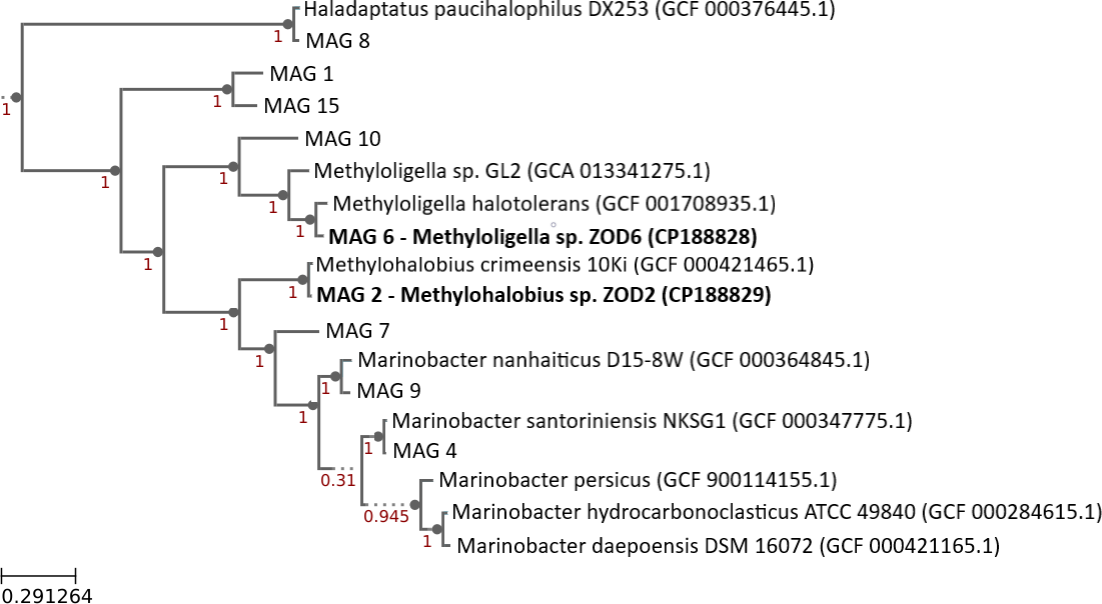
Phylogenetic tree showing the placement of the nine MAGs recovered from the Zodletone methanotrophic enrichment. The two MAGs described in this study, Methylohalobius sp. ZOD2 (CP188829) and Methyloligella sp. ZOD6 (CP188828), are shown in bold with their respective GenBank accession numbers. The tree was generated using the “Insert Genome Into SpeciesTree” tool (v2.2.0) based on conserved bacterial marker genes, with nine neighboring reference genomes included for context. Branch support values are shown at nodes. The scale bar represents substitutions per site.

To determine the community composition, total DNA was isolated using the FastDNA SPIN Kit for Soil (MP Biomedicals, USA). Paired-end shotgun metagenomic sequencing (2 × 150  bp) was performed on an Illumina Novaseq X platform (Illumina, Inc., San Diego, CA, USA) at Biomarker Technologies (BMKGENE) USA Inc. A library was constructed through enzymatic fragmentation and PCR amplification using the VAHTS Universal Plus DNA Library Prep Kit (Vazyme, China). Raw reads were trimmed, removing adaptors and low-quality sequences with fastp v0.24.3 (3), resulting in 72,203,262 clean reads totaling 11,004,544,590 bp. These clean reads were assembled using MEGAHIT v1.2.9 (4). Scaftigs were aligned using Bowtie2 v2.5.4 (5), and ORFs predicted with MetaGeneMark v3.26 (6). Taxonomy was inferred using MEGAN v6.25.10 and the microNR database (release date: February 2022) (7). MAGs were binned with MetaBAT2 v1.7 (8) and MaxBin2 v2.2.4 (9), refined with DASTool v1.1.2 (10), and assessed using CheckM v1.0.18 (11). High-quality bins were annotated with RASTtk v1.073 (12), classified using GTDB-Tk v1.7 (13), and placed phylogenetically via SpeciesTree v2.2 (Fig. 1) (14) using 49 core COGs and 9 public genomes. Functional annotation was conducted using DRAM v0.1.2 (15). Default parameters were used for all software, unless stated otherwise.

Of the nine high-quality MAGs, MAG 2 and M AG 6 encode genes for methane metabolism. MAG 2, identified as a close member of the bacterium, *Methylohalobius crimeensis* (16), and MAG 6, identified as *Methyloligella* sp., and reads were assembled into near-complete genomes (96.16% complete/1.03% contaminated and 99% complete/1.14% contaminated, respectively). Average nucleotide identity (ANI, determined by GTDB-Tk) to their closest references, *Methylohalobius crimeensis* (GCF_000421465.1) and *Methyloligella halotolerans* (GCF_001708935.1) (17), was 95.47% for MAG 2 and 83.73% for MAG 6, indicating that MAG 2 represents a new *Methylohalobius* species and MAG 6a potentially novel *Methyloligella* species. We therefore designate them as *Methylohalobius* sp. strain ZOD2 and *Methyloligella* sp. strain ZOD6. Comparative features are summarized in Table 1. Annotation via Prokka v1.14.5 (18) and metabolic pathway reconstruction by KEGG mapper (19) revealed that strain ZOD2 encodes the particulate form of methane monooxygenase, which is lacking in strain ZOD6. Methylotrophs that oxidize methane under saline conditions play a crucial role in reducing greenhouse gas emissions from AOG and PWST.

**TABLE 1:** Genomic features of the two methylotrophic MAGs recovered from the Zodletone methanotrophic enrichment annotated by Prokka and NCBI Prokaryotic Genome Annotation Pipeline (PGAP) (20)

MAG name	*Methylohalobius* sp. ZOD2	*Methyloligella* sp. ZOD6
Biosample accession No.	SAMN47862349	SAMN47862350
GenBank accession No.	CP188829	CP188828
CheckM completeness (%)	96.16	99
CheckM contamination (%)	1.03	1.14
No. of contigs	66	26
Genome size (bp)	3,233,401	2,998,024
N50 (bp)	80,659	317,859
G + C%	58	63.1
Total CDS	3,067	2,789
Total genes	3,112	2,837
RNA genes	45	48
No. of tRNA	41	43
No. of ncRNA	4	4
No. of 5S rRNA	0	1
No. of 16S rRNA	1	0
No. of 23S rRNA	0	1

## Data Availability

The draft genome sequences are available in the NCBI database under BioProject accession number PRJNA1248234. The GenBank accession numbers for the two MAGs are CP188829 and CP188828 and were annotated using the NCBI Prokaryotic Genome Annotation Pipeline. The raw sequencing reads have been deposited in the Sequence Read Archive (SRA) under accession number SRP577458.
